# Intention to Delay Retirement and Its Influencing Factors Among Near‐Retirement Nurses: A Cross‐Sectional Study

**DOI:** 10.1155/jonm/6063712

**Published:** 2026-05-10

**Authors:** Jianmei Chen, Fen Yang, Suiwen Liu, Zeshan Tan, Chunyan Ye, Jiemei Gao, Sha Ding, Minjuan Zhou

**Affiliations:** ^1^ Department of Nursing, Guangzhou Chest Hospital Affiliated to Guangdong Pharmaceutical University, Guangzhou, Guangdong, China

**Keywords:** cross-sectional studies, intention, nurses, nursing workforce, retirement

## Abstract

**Background:**

The ageing nursing workforce poses a growing challenge to healthcare systems worldwide. Delaying retirement among experienced nurses has been proposed as a potential workforce strategy, yet empirical evidence from China remains limited, particularly from a management‐oriented perspective under ongoing retirement‐age reforms.

**Aim:**

To examine the prevalence of delayed retirement intention and to identify individual, occupational, and organisational factors associated with such intention among near‐retirement nurses in Guangzhou, China.

**Methods:**

A theory‐informed cross‐sectional survey was conducted from May to August 2025 among 298 nurses aged ≥ 45 years from 37 hospitals in Guangzhou. Guided by an integrated push–pull and job demands–resources framework, a structured questionnaire measured sociodemographic and work characteristics, economic and family stress, job satisfaction and occupational burnout. Data were analysed using descriptive statistics, nonparametric tests, Spearman correlation and ordinal logistic regression.

**Results:**

Only 13.4% of near‐retirement nurses expressed willingness to delay retirement, whereas 38.3% were unwilling. In multivariable ordinal logistic regression, contract‐based employment, higher professional title, greater economic and family stress and lower occupational burnout were independently associated with higher levels of delayed retirement intention.

**Conclusion:**

Near‐retirement nurses demonstrated a generally low intention to delay retirement. Organisational and employment‐related factors were prominently associated with delayed retirement intention, underscoring the importance of work‐system conditions in shaping late‐career retention.

**Implications for Nursing Management:**

The findings suggest that modifiable organisational factors—such as occupational burnout, late‐career role design and employment arrangements—are associated with delayed retirement intention. These areas may represent potential points of attention for nursing management when considering strategies to support the retention of experienced nurses under retirement‐age reform.

## 1. Introduction

The rapid ageing of the nursing workforce has become a critical challenge to healthcare systems worldwide [1]. By 2030, the supply of healthcare workers is projected to be 65 million, falling short of the estimated demand of 80 million, with nurses and midwives representing the largest shortfall [1]. In developed countries such as the UK and Australia, over one‐third of registered nurses are aged over 50, and this proportion continues to rise [2]. The retirement of experienced nurses aggravates workforce shortages, threatens care quality and continuity, and results in the loss of valuable mentoring and organisational knowledge [3].

China is experiencing a similar crisis, with a rapidly ageing nursing workforce and a statutory retirement age of 55 for female and 60 for male nurses, which is relatively low compared to many Western countries [4]. Notably, more than 97% of nurses in China are female [4], and under the traditional statutory retirement system, women generally reached retirement eligibility earlier than men, with a five‐year difference in many occupational groups (55 vs. 60). In response to population ageing and workforce shortages, China formally initiated a nationwide, phased reform to gradually raise the statutory retirement age for both men and women from 1 January 2025 [5].

This unique demographic and policy landscape may further complicate workforce sustainability, particularly as gender differences in retirement intentions may be amplified under such conditions. Although the number of registered nurses has increased, there remains a significant shortage of clinical nursing staff, especially in large cities and specialised hospitals [6]. Prior to the formal statutory reform, delayed retirement had been widely discussed and explored through policy debates and local initiatives in China. However, empirical evidence shows that Chinese nurses’ intention to delay retirement remains low. Surveys in Shandong and Jiangsu provinces found that more than 70% of mid‐ and senior‐career nurses are unwilling to postpone retirement, mainly due to health concerns, family caregiving responsibilities and dissatisfaction with current policies [7, 8]. Both cross‐sectional and qualitative studies demonstrate that factors such as health status, family support, job satisfaction, organisational climate and policy awareness significantly influence retirement intentions [6, 9, 10].

International evidence confirms that nurses’ retirement decisions are shaped by a mix of “push” (ill health, workplace stress and family needs) and “pull” (financial need, professional value and flexible work options) factors [2, 11]. Evidence suggests that supportive organizational policies and flexible employment opportunities may help retain older nurses, yet robust interventions are still lacking, especially in the Chinese context [3]. Despite growing interest, large‐sample, multicentre studies focusing on delayed retirement intention among Chinese nurses remain rare. There is an urgent need for robust research to understand prevalence and influencing factors, especially in major urban centres.

### 1.1. Conceptual Framework

To provide a management‐oriented theoretical foundation, this study is grounded in an integrated push–pull framework of retirement decision‐making and the job demands–resources (JD‐R) model. The push–pull framework conceptualises retirement decisions as the result of opposing forces, whereby “push” factors drive individuals out of the workforce, while “pull” factors motivate continued labour participation. This perspective has been widely discussed since the foundational review by Beehr [12] and further elaborated in subsequent psychological models of retirement decision‐making [13].

In parallel, the JD‐R model, originally proposed by Demerouti et al. [14] and further developed by Bakker and Demerouti [15], provides a robust management‐oriented framework for understanding how job characteristics influence employee wellbeing and retention. According to the JD‐R model, excessive job demands deplete employees’ physical and psychological resources, leading to burnout and withdrawal intentions, whereas job resources foster motivation, engagement, and sustained work participation.

In the context of near‐retirement nurses, push factors may include occupational burnout, declining health status and family caregiving responsibilities, which increase strain and reduce work sustainability. In contrast, pull factors may encompass economic and family financial stress, professional role and status and employment‐related incentives that encourage continued labour participation. From a JD‐R perspective, high job demands contribute to burnout and reduced capacity to remain in work, while job resources—such as job satisfaction, professional recognition and flexible employment arrangements—may buffer these demands and enhance nurses’ willingness to remain in the workforce beyond the statutory retirement age.

**FIGURE 1:**
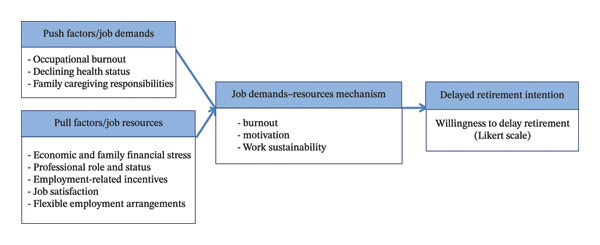
Conceptual framework of delayed retirement intention among near‐retirement nurses based on the integrated push–pull and job demands–resources (JD‐R) models. Note: The push–pull framework conceptualises retirement decisions as the result of opposing forces that either drive individuals out of the workforce (push factors) or encourage continued labour participation (pull factors). Within the job demands–resources (JD‐R) model, push factors are conceptualised as job demands that increase strain and burnout, whereas pull factors are conceptualised as job resources that enhance motivation, work sustainability and retention.

Accordingly, this study integrates the push–pull framework and the JD‐R model to guide the selection of study variables, the analytical strategy and the interpretation of findings. Figure 1 presents the conceptual model, illustrating how individual, occupational and organisational factors interact to influence delayed retirement intention among near‐retirement nurses.

Guided by this integrated theoretical framework, the present study aimed to examine the prevalence of delayed retirement intention and to identify individual, occupational and organisational factors associated with such intention among near‐retirement nurses in Guangzhou, China. The findings are intended to inform nursing management strategies and policy development under delayed retirement reforms.

## 2. Methods

### 2.1. Study Design and Participants

This cross‐sectional study followed the Strengthening the Reporting of Observational Studies in Epidemiology (STROBE) checklist for cross‐sectional studies. Data were collected between May and August 2025 from 37 hospitals in Guangzhou, China, including tertiary hospitals, specialised hospitals and community health service centres. Eligible participants were registered nurses aged ≥ 45 years, within five years of the statutory retirement age, with ≥ 20 years of nursing experience and continuously engaged in clinical practice for at least the past year. Nurses on long‐term leave, with severe cognitive or psychiatric disorders, or who had formally applied for retirement were excluded.

The sample size was determined based on methodological recommendations for regression modelling. Previous simulation studies suggest that a minimum of 10 outcome events per predictor variable (EPV) is required to ensure model stability and reliable parameter estimation in logistic‐type regression models [16]. This principle is commonly extended to ordinal logistic regression to ensure adequate observations relative to the number of estimated parameters [17]. Considering the number of key demographic, health‐related, and job‐related variables included in the final model, as guided by the conceptual framework, a sample size of 298 participants was considered adequate for the planned analyses.

Of 304 questionnaires distributed via the Wenjuanxing online survey platform, 298 valid responses were obtained (response rate: 98%). Ethical approval was granted by the Ethics Committee of Guangzhou Chest Hospital (KY‐2025‐048), and completion of the anonymous questionnaire was considered implied consent.

### 2.2. Measures

The primary outcome was delayed retirement intention, measured using a single‐item question: “Are you willing to delay your retirement?”, rated on a five‐point Likert scale (1 = very unwilling to 5 = very willing) [13].

Single‐item measures have been widely used in retirement and occupational research to capture global intention constructs, particularly when the concept is relatively concrete, unidimensional and forward‐looking. Prior studies suggest that single‐item indicators of intention demonstrate acceptable validity and predictive utility in large‐scale survey research.

Nevertheless, we acknowledge that the use of a single‐item measure precludes the assessment of internal consistency and may not fully capture the multidimensional and dynamic nature of retirement decision‐making. Accordingly, associations and effect sizes related to delayed retirement intention are interpreted with appropriate caution.

Explanatory variables included:

 Demographic and work‐related characteristics: age, gender, education, professional title, employment type, years of experience and workplace.
 Occupational burnout: measured using the Chinese version of the 9‐item Maslach Burnout Inventory‐Human Services Survey [18], covering emotional exhaustion, depersonalisation and reduced personal accomplishment (Cronbach’s *α* = 0.864).
 Job Satisfaction: Job satisfaction was assessed using a four‐item scale developed by the research team, covering work environment, leadership support, workload, and career development. Item development was informed by existing literature and expert consultation in nursing management. A pilot test with 38 nurses was conducted to refine item wording and response options. The scale demonstrated good internal consistency in the present sample (Cronbach’s *α* = 0.885).
 Health status: self‐reported overall health and perceived work ability.
 Economic and Family Stress: Economic and family stress was measured using a five‐item scale assessing perceived financial burden related to housing, children’s education and medical expenses. This scale was developed for exploratory purposes based on prior studies and expert input. Internal consistency was acceptable but modest (Cronbach’s *α* = 0.673), suggesting heterogeneity in the construct. Accordingly, findings related to economic and family stress should be interpreted with caution, and further validation is warranted in future research.

### 2.3. Statistical Analysis

Data were analysed using SPSS version 26. Descriptive statistics were used to summarise participant characteristics. Due to non‐normal distributions, Mann–Whitney U and Kruskal–Wallis H tests were applied for univariate comparisons, and Spearman’s rank correlation was used to examine associations among continuous variables.

Variable selection for the ordinal logistic regression model was guided by the integrated push–pull and JD‐R theoretical framework, supplemented by univariate screening (*p* < 0.10). Demographic variables (e.g., age), health‐related factors, and key job demands and resources were considered for inclusion based on theoretical relevance. Importantly, the integrated push–pull and JD‐R framework was not used solely for variable categorisation. It also informed the analytical strategy by distinguishing background characteristics (e.g., age and health) from theoretically central job demands and job resources. In line with the JD‐R model, variables representing job demands (e.g., occupational burnout) and job resources or employment‐related incentives (e.g., employment type, professional title, job satisfaction and economic/family stress) were simultaneously entered into the multivariable model to examine their relative contributions to delayed retirement intention. This approach allows the analysis to reflect the JD‐R proposition that job demands and resources jointly shape work‐related outcomes, including retention and withdrawal intentions.

Ordinal logistic regression was conducted to identify factors associated with delayed retirement intention. The proportional odds assumption was tested using the test of parallel lines and was satisfied (*p* > 0.05). Multicollinearity among predictors was assessed using variance inflation factors (VIF), with all values below the commonly accepted threshold, indicating no serious multicollinearity.

Odds ratios (OR) with 95% confidence intervals (CI) were reported. Statistical significance was set at *p* < 0.05 (two‐sided).

## 3. Results

Of the 304 distributed questionnaires, 298 were valid (response rate: 98%). Most participants were female (98.0%), held the title of charge nurse or above (87.6%), and worked in frontline clinical positions (75.2%). Given that only six male nurses were included, gender comparisons should be interpreted with caution because estimates may be statistically unstable. Accordingly, gender was not treated as a focal explanatory factor, and its association is reported descriptively only. The majority of participants were permanently employed (84.2%) and aged 45–54 years (96.3%). The mean years of service was 29.1 years (SD = 4.2). Detailed demographic and work‐related characteristics are shown in Table 1.

**TABLE 1:** Demographic and work‐related characteristics of the participants (*N* = 298).

Variable	Category	*n*	%
Gender	Male	6	2.0
	Female	292	98.0

Professional title	Nurse	7	2.3
	Senior nurse	30	10.1
	Charge nurse	177	59.4
	Deputy Chief Nurse and above	84	28.2

Current position	Clinical frontline	224	75.2
	Management	39	13.1
	Teaching/research	1	0.3
	Others^a^	34	11.4

Employment type	Permanent	251	84.2
	Contract‐based	47	15.8

Chronic illness or health problem	Yes	82	27.5
	No	216	72.5

Primary source of income	Own salary	296	99.3
	Pension	2	0.7

Need to care for family members	Yes	238	79.9
	No	60	20.1

Hospital grade	Tertiary A	220	73.8
	Tertiary B	32	10.7
	Secondary A	22	7.4
	Secondary B	1	0.3
	Community Hospital	10	3.4
	Others^b^	13	4.4

Age group (years)	45–49	158	53.0
	50–54	129	43.3
	≥ 55	11	3.7

Years of service	20–29	155	52.0
	30–34	118	39.6
	≥ 35	23	7.7

^a^Others: vaccination, public health, nursing administration, supply room, inspection room.

^b^Other hospital types.

### 3.1. Delayed Retirement Intention and Health Status

Among the 298 nurses, 38.3% reported being “very unwilling” and 29.2% “unwilling” to delay retirement, while 13.4% expressed willingness (including “willing” and “very willing”). Most respondents rated their health as average or above (85.3%), although 14.8% reported poor or very poor health. Regarding perceived work ability, 68.8% considered themselves fully or basically competent to continue working, whereas 31.2% felt barely competent or unable to work due to health reasons. Detailed distributions are presented in Table 2.

**TABLE 2:** Distribution of delayed retirement intention and health status (*N* = 298).

Item	*n*	%
Delayed Retirement Intention		
Very unwilling	114	38.3
Unwilling	87	29.2
Neutral	57	19.1
Willing	33	11.1
Very willing	7	2.3
Self‐Rated Health Status		
Very good	27	9.1
Good	75	25.2
Average	152	51.0
Poor	39	13.1
Very poor	5	1.7
Health Support for Work Competence		
Fully competent	64	21.5
Basically competent	141	47.3
Barely competent	82	27.5
Unable to perform	11	3.7

### 3.2. Scale Scores

The mean (±SD) scores were 23.86 ± 7.56 for occupational burnout, 16.94 ± 3.95 for economic and family stress, and 12.61 ± 3.01 for job satisfaction. Score ranges were 9–44, 8–28, and 4–20, respectively (Table 3).

**TABLE 3:** Scores of occupational burnout, economic and family stress and job satisfaction.

Indicator	Mean ± SD	Min	Max
Occupational burnout	23.86 ± 7.56	9	44
Economic and family stress	16.94 ± 3.95	8	28
Job satisfaction	12.61 ± 3.01	4	20

### 3.3. Univariate Analysis

Univariate analyses showed that willingness to delay retirement differed across several demographic and work‐related characteristics (*p* < 0.05). Although a statistically significant gender difference was observed, this finding should be interpreted cautiously due to the extremely small number of male participants (*n* = 6) and is considered descriptive rather than inferential.

Willingness to delay retirement was significantly higher among nurses aged ≥ 50 years, those with longer service, higher educational attainment or higher professional titles (all *p* < 0.05). Management staff and contract‐based nurses also expressed greater willingness compared with frontline or permanently employed nurses. Better self‐rated health, higher perceived work competence, lower occupational burnout, greater economic and family stress and higher job satisfaction were all positively associated with delayed retirement intention (all *p* < 0.05) (Table 4).

**TABLE 4:** Univariate analysis of factors associated with delayed retirement intention among nurses (*N* = 298).

Variable	Group	*n*	Mean rank	Test	df	*p*
Gender	Male	6	74.25	*U* = 424.5		**0.024**
	Female	292	151.05			

Age group	45–49	158	137.23	*χ* ^2^ = 8.71	2	**0.013**
	50–54	129	161.11			
	≥ 55	11	189.64			

Years of service	20–29	157	135.72	*χ* ^2^ = 9.41	2	**0.009**
	30–34	118	165.78			
	≥ 35	23	160.02			

Education	Diploma or below	9	124.5	*χ* ^2^ = 8.22	3	**0.036**
	Associate	92	131.43			
	Bachelor	193	158.65			
	Postgraduate	4	180.13			

Title	Nurse	7	177.07	*χ* ^2^ = 29.78	3	**< 0.001**
	Senior Nurse	30	101.32			
	Supervisor Nurse	177	139.5			
	Assoc. Chief or above	84	185.48			

Post	Clinical	224	138.73	*χ* ^2^ = 21.00	3	**< 0.001**
	Management	39	199.87			
	Teaching/Research	1	57.5			
	Other	34	165.41			

Employment type	Permanent	251	143.25	*U* = 4328.5		**0.002**
	Contract	47	182.9			

Chronic disease	Yes	82	126.28	*U* = 6952.0		**0.003**
	No	216	158.31			

Family care	Yes	238	149.26	*U* = 7083.0		0.920
	No	60	150.45			

Hospital level	Tertiary A	220	150.45	*χ* ^2^ = 11.55	4	**0.015**
	Tertiary B	32	108.13			
	Secondary A	22	130.68			
	Secondary B	1	220.5			
	Community	10	109.95			

Overall health	Very good	27	212.76	*χ* ^2^ = 44.48	4	**< 0.001**
	Good	75	173.79			
	Average	152	142.41			
	Poor	39	90.27			
	Very poor	5	121.1			

Work competence	Fully competent	64	206.75	*χ* ^2^ = 70.01	3	**< 0.001**
	Basically competent	141	157.84			
	Barely competent	82	99.14			
	Unable	11	84.91			

Burnout	≤ 19	99	193.6	*χ* ^2^ = 63.23	2	**< 0.001**
	20–26	98	154.41			
	≥ 27	101	101.5			

Economic/family stress	≤ 15	101	132.87	*χ* ^2^ = 21.77	2	**< 0.001**
	16–18	99	135.07			
	≥ 19	98	181.22			

Job satisfaction	≤ 12	158	127.9	*χ* ^2^ = 25.99	2	**< 0.001**
	13–14	55	159.45			
	≥ 15	85	183.21			

*Note:* Bold values indicate *p* < 0.05.

### 3.4. Multivariate Ordinal Logistic Regression

The proportional odds assumption was tested using the test of parallel lines and was satisfied, supporting the use of ordinal logistic regression. The final model showed a significant improvement over the intercept‐only model (−2 Log Likelihood = 619.32 vs. 807.73; *χ* ^2^ = 188.40, df = 33, *p* < 0.001).

Goodness‐of‐fit statistics indicated satisfactory model fit (Pearson *χ* ^2^ = 1031.22, df = 1087, *p* = 0.886; deviance *χ* ^2^ = 607.99, df = 1087, *p* = 1.000). Pseudo *R* ^2^ values suggested moderate explanatory power (Nagelkerke *R* ^2^ = 0.501; McFadden *R* ^2^ = 0.230).

Multicollinearity among independent variables was assessed using VIF. VIF values ranged from 1.03 to 3.06, indicating no evidence of problematic multicollinearity among predictors.

In the final ordinal logistic regression model, contract‐based employment, higher professional title, greater economic and family stress and lower occupational burnout were independently associated with a higher willingness to delay retirement (all *p* < 0.05). Lower occupational burnout was statistically associated with higher reported levels of delayed retirement intention.

Given the extreme gender imbalance in the sample, gender was excluded from the final multivariate model to avoid statistically unstable parameter estimates. A sensitivity analysis comparing models with and without gender yielded highly consistent results, indicating that the observed associations were robust and not driven by gender distribution. Detailed results are presented in Table 5.

**TABLE 5:** Ordinal logistic regression of factors associated with delayed retirement intention.

Variable	Comparison	OR (95% CI)	*p* value
Employment type	Permanent (Ref) vs Contract	0.30 (0.15, 0.59)	0.001

Professional title	Nurse‐in‐charge vs Dep. Chief Nurse+	0.12 (0.04, 0.36)	< 0.001
	Charge Nurse vs Dep. Chief Nurse+	0.44 (0.24, 0.81)	0.008

Economic/family stress	Low vs High (Ref)	0.22 (0.12, 0.41)	< 0.001
	Medium vs High (Ref)	0.38 (0.22, 0.68)	0.001

Occupational burnout	Low vs High	4.90 (2.28, 10.54)	< 0.001
	Medium vs High	2.64 (1.35, 5.13)	0.004

*Note:* Ref = reference category.

## 4. Discussion

Only 13.4% of near‐retirement nurses in Guangzhou expressed willingness to postpone retirement, indicating a generally low intention to delay retirement in this population. Multivariate analysis indicated that contract‐based employment, higher professional title, greater economic and family stress and lower occupational burnout were statistically associated with higher levels of delayed retirement intention. This study found that only a small proportion of near‐retirement nurses were willing to delay retirement. Employment type, professional title, economic and family stress and occupational burnout were all associated with this intention.

### 4.1. Comparison With Previous Studies

The low proportion of nurses in our study willing to delay retirement aligns with patterns reported both in China and internationally. National surveys in China have found that over 70% of senior nurses do not wish to extend their employment, most often due to health problems and family caregiving responsibilities [7, 8]. Similar trends have been documented in the United Kingdom, Australia and the United States, where declining health, rising workload, and insufficient organisational support contribute to retirement at or before the statutory age [2, 11]. Our results add to this body of evidence, highlighting that retirement intentions among nurses are influenced by a multifaceted combination of personal, professional and organisational factors.

### 4.2. Employment Type and Economic Stress

In this study, contract‐based nurses reported a greater intention to delay retirement compared with permanently employed staff. This pattern may reflect differences in perceived job insecurity and financial pressure commonly reported among contract employees, as documented in both Chinese and international research [6, 11]. Employment status has been consistently associated with differences in retirement planning, with contract workers more likely to extend their careers due to lower pension benefits and limited social security coverage. The observed association with economic and family stress may reflect the influence of financial considerations. However, given the exploratory nature of this measure, further validation with more robust instruments is needed.

### 4.3. Professional Title, Occupational Burnout and Job Satisfaction

Nurses with higher professional titles in this study were more willing to delay retirement, aligning with previous findings that career advancement, professional recognition and expanded decision‐making roles can motivate extended workforce participation [7, 9]. Lower occupational burnout was independently associated with higher reported levels of delayed retirement intention, while higher job satisfaction showed a positive association in univariate analysis. These results are consistent with evidence that supportive work environments, opportunities for skill development and manageable workloads promote nurse retention and delay retirement [3, 6, 10]. These patterns are consistent with previous evidence showing that work environment, workload and professional support are closely related to nurse retention and retirement decisions.

### 4.4. Health, Family Responsibilities and Policy Implications

In this study, better self‐rated health and higher perceived work competence were positively associated with the intention to delay retirement, whereas chronic illness and family caregiving responsibilities were negatively associated with delayed retirement intention. These findings are consistent with both Chinese and international evidence indicating that physical health status and caregiving demands are closely associated with retirement decisions among nurses [10, 11]. With China progressively implementing policies to extend the statutory retirement age for both men and women [5], healthcare organisations must proactively address these challenges. These findings highlight the potential importance of health status and caregiving responsibilities in shaping retirement decisions, particularly in the context of ongoing retirement‐age reform in China.

### 4.5. Incremental Contribution

This study contributes to the nurse‐retirement literature by extending predominantly descriptive evidence toward a more management‐oriented understanding of delayed retirement intention within the context of China’s ongoing retirement‐age reform. While prior international work has consistently shown that retirement timing is shaped by a mix of personal and organisational determinants [2, 3], and Chinese research has examined retirement planning and work‐related variables among older nurses [19], less is known about how these factors combine under China’s current retirement‐age reform context.

By applying an integrated push–pull and JD‐R framework, our findings suggest that intention to delay retirement is not only a function of individual health or family circumstances but is also closely associated with employment arrangements and work‐system conditions. By structuring the analysis according to the JD–R framework, this study highlights how job demands (e.g., burnout) and job resources (e.g., employment arrangements and professional roles) may operate together in shaping delayed retirement intention.

Notably, the coexistence of generally adequate self‐rated health with low willingness to delay retirement suggests that improving “fitness to work” alone may be insufficient; organisational and employment‐related conditions may play an important role in shaping retention strategies in the near‐retirement segment, consistent with broader evidence that organisational context and perceived work sustainability are central in older nurses’ retirement decisions [2, 20].

### 4.6. China‐Specific Mechanisms

China‐specific mechanisms under retirement‐age reform. The Chinese nursing workforce is highly feminised, with women constituting the vast majority of nurses [4], and retirement decisions occur within a policy environment historically characterised by gender‐differentiated statutory retirement ages and rapid population ageing. Against this backdrop, China has now formally initiated a phased national reform to gradually raise the statutory retirement age beginning 1 January 2025 [5]. These structural features plausibly amplify the salience of “employment security” and “returns to continued work” for near‐retirement nurses. For example, differential benefit expectations, perceived pension adequacy, and institutional employment protections may shape whether delayed retirement is viewed as a feasible bridge or as an undesirable extension of strain—especially when late‐career nurses face accumulated workloads, physical demands and role saturation [19]. Our results, therefore, point to a context‐specific mechanism: under a reform trajectory that extends working lives, organisational arrangements (e.g., contract vs. permanent employment) and job‐system experiences (burnout vs. sustainable work design) may become stronger signals for the desirability and practicality of staying beyond what is captured by generic correlates reported across settings [2, 3, 20].

### 4.7. Management Implications

Given the cross‐sectional design, the following management implications are derived from observed associations rather than causal effects. Delayed retirement among near‐retirement nurses in this study appears to be more closely related to workforce design and employment conditions than to individual motivation alone.

From a JD‐R perspective, the findings of this study suggest that both job demands and job resources are associated with delayed retirement intention. In particular, the observed association between lower occupational burnout and higher willingness to delay retirement suggests that excessive job demands may play a role in shaping retirement intentions. These findings point to several areas that may warrant attention in practice, including staffing levels, workload distribution, ergonomic support and scheduling arrangements in late‐career nursing roles.

In addition, the associations with employment type and professional title suggest that organisational and career‐related factors may also influence retirement intentions, pointing to potential areas for review in employment arrangements and role design.

The observed association between economic and family stress and delayed retirement intention may reflect the role of financial considerations. However, this relationship should be interpreted with caution and warrants further investigation using more robust measures.

Taken together, this study contributes to reframing delayed retirement among nurses as a management and work‐system phenomenon. By linking specific empirical findings to potential organisational strategies, the results provide a context‐specific perspective on how organisational and work‐related factors are associated with delayed retirement intention.

### 4.8. Strengths and Limitations

This study has several strengths. First, its multicentre design and relatively large sample size enhance the robustness of the findings within the Guangzhou nursing workforce. Second, the comprehensive assessment of sociodemographic, occupational, and psychosocial variables allows for a nuanced examination of the multifactorial influences on delayed retirement intention.

Several limitations should also be acknowledged. First, the cross‐sectional design precludes causal inference, and longitudinal studies are needed to examine how retirement intentions evolve over time. Second, the sample was drawn exclusively from hospitals in Guangzhou, which may limit the generalisability of the findings to other regions with different healthcare systems or workforce structures.

Third, delayed retirement intention was measured using a single‐item indicator. Although this approach has been widely used in retirement and occupational research to assess global intentions, it may not fully capture the multidimensional and dynamic nature of retirement decision‐making, and measurement error cannot be ruled out. Accordingly, effect sizes and associations related to delayed retirement intention should be interpreted with appropriate caution.

Finally, reliance on self‐reported measures introduces the potential for recall bias and social desirability bias, which may affect the accuracy of responses. Future research incorporating objective indicators and broader geographic sampling would strengthen external validity.

### 4.9. Implications for Practice and Future Research

The findings of this study underscore the urgent need for evidence‐informed strategies to retain experienced nurses as China navigates the challenges of an ageing workforce. Health service leaders and policymakers should prioritise measures that strengthen employment security, address gender‐ and age‐related disparities in retirement policy, promote healthy ageing through workplace wellness programmes and foster organisational cultures that enhance job satisfaction and reduce burnout. Flexible work arrangements and tailored retention initiatives may be particularly effective for nurses approaching statutory retirement age.

From a research perspective, longitudinal studies are needed to track the evolution of retirement intentions over time and assess the causal pathways underlying observed associations. Qualitative inquiry could provide deeper insight into the lived experiences and decision‐making processes of near‐retirement nurses, complementing quantitative findings. Moreover, future evaluations should examine the impact of China’s phased retirement‐age reform and related policy interventions on nurse retention, workforce sustainability, and patient care quality.

## 5. Conclusion

This multicentre cross‐sectional study found a generally low level of willingness to delay retirement among near‐retirement nurses. Employment type, professional title, economic and family stress and occupational burnout were associated with delayed retirement intention.

These findings indicate that delayed retirement intention is related to organisational and work‐related conditions, in addition to individual factors. The results contribute to a better understanding of factors associated with late‐career workforce participation among nurses.

## Funding

This research received no specific grant from funding agencies in the public, commercial, or not‐for‐profit sectors.

## Disclosure

The study was performed as part of the employment of the authors at Guangzhou Chest Hospital.

## Conflicts of Interest

The authors declare no conflicts of interest.

## Data Availability

The datasets generated and analyzed during the current study are available from the corresponding author on reasonable request. No other publications or manuscripts using these data are currently under consideration elsewhere.
